# IgA vasculitis update: Epidemiology, pathogenesis, and biomarkers

**DOI:** 10.3389/fimmu.2022.921864

**Published:** 2022-10-03

**Authors:** Liyun Xu, Yongzhen Li, Xiaochuan Wu

**Affiliations:** Department of Pediatrics, The Second Xiangya Hospital, Central South University, Changsha, China

**Keywords:** IgA vasculitis, epidemiology, immunopathogenesis, genetic factors, biomarkers

## Abstract

Immunoglobulin A vasculitis (IgAV), formerly known as Henoch-Schönlein purpura, is the most common systemic vasculitis in children, characterized by diverse clinical manifestations with a wide spectrum ranging from isolated cutaneous vasculitis to systemic involvement. The incidence of IgAV is geographically and ethnically variable, with a prevalence in autumn and winter, suggesting a driving role that genetic and environmental factors play in the disease. Although IgAV has a certain degree of natural remission, it varies widely among individuals. Some patients can suffer from severe renal involvement and even progress to end-stage renal disease. Its pathogenesis is complex and has not been fully elucidated. The formation of galactose-deficient IgA1 (Gd-IgA1) and related immune complexes plays a vital role in promoting the occurrence and development of IgAV nephritis. In addition, neutrophil activation is stimulated through the binding of IgA to the Fc alpha receptor I expressed on its surface, resulting in systemic vascular inflammation and tissue damage. Starting from the epidemiological characteristics, this article will review the role of immunological factors such as Gd-IgA1, autoantibodies, circulating immune complexes, complement system, cellular immunization, and the contributions of environmental and genetic factors in the pathogenesis of IgAV, and conclude with the major biomarkers for IgAV.

## 1 Introduction

Immunoglobulin A vasculitis (IgAV), also referred to as Henoch-Schönlein purpura, is the most common primary vasculitis in childhood. The 2012 Chapel Hill Conference defined it as a vasculitis with IgA1-dominant immune deposits, affecting small vessels (predominantly capillaries, venules, or arterioles), highlighting the role of IgA in the disease (1). According to different clinical presentations, it can be divided into skin-limited IgA vasculitis (cutaneous IgA-dominant vasculitis without detectable involvement of systemic organs, purely manifested as non-thrombocytopenic purpura) and systemic form (present in at least one organ in addition to skin, e.g., often combined with joint, gastrointestinal and renal involvement, a few involves pulmonary and central nervous system) (2). The course of IgAV is mostly self-limiting. However, there is a recurrence rate of one-third for pediatric patients (3). Renal involvement is the prominent determinant of prognosis, since up to 20%–80% of children showing signs of nephritis such as hematuria and/or proteinuria within four to six weeks at the initial consultation, and 1%–7% of children with IgAV nephritis (IgAVN) may progress to renal failure or end-stage renal disease (ESRD) (4, 5). The risk of progression to chronic kidney disease in patients with ≥50% glomerular crescents on biopsy ranges from 5% to 20% (5). In addition, recent studies have shown that patients with tubular or interstitial lesions and glomerulosclerosis are associated with poor renal outcomes, and patients with both nephritic and nephrotic syndrome have the highest risk for progression to ESRD (6–9). Adult patients face a poorer prognosis than children, leaning more towards a protracted and chronic course, of who the incidence of ESRD is up to 32% (10–12).

IgAVN and IgA nephropathy (IgAN) have similar features, both characterized by hematuria, proteinuria, and the glomerular mesangium deposition of immune complexes, sharing a common pathogenetic basis. Some scholars have even suggested that IgAVN and IgAN should be classified as the same disease, but IgAN is confined to kidney manifestations and usually begins with asymptomatic hematuria. Even if IgA deposits occur in extrarenal sites in some patients but no corresponding clinical manifestations, renal biopsy remains essential for making a definitive diagnosis; additionally, there is no seasonal variation in the incidence of IgAN. It tends to be more frequent in children over 10 years of age and adults; The prognosis is quite different between the two. IgAN has a far worse outcome than IgAVN, with 30%–40% of patients reaching ESRD 20–30 years after first clinical presentation (13), while some IgAVN patients with proteinuria are occasionally transient and seem to have a tendency to resolve spontaneously. All these discrepancies determine that the treatment strategies and follow-up goals of the two diseases are distinct.

On the other hand, although patients with mild urinary abnormalities in IgAVN are also at risk of developing permanent kidney damage, severe cases of IgAV can manifest as acute kidney injury, even rapid deterioration of renal function similar to rapidly progressive glomerulonephritis, or macroscopic hematuria and chronic kidney damage. At the same time, life-threatening complications such as severe gastrointestinal bleeding, intestinal intussusception, or perforation, can also occur in the acute phase of IgAV, which require clinicians to pay substantial attention (14). Therefore, it is of great significance to study the pathogenesis of IgAV and learn how to identify severe cases or patients at risk of chronic kidney injury at an early stage, which will be conducive to clinically targeted early prevention and treatment.

Efforts have been made to further understand IgAV. Some scholars have recently reviewed the role of genetic and geographical factors and galactose-deficient IgA1 (Gd-IgA1) in the pathogenesis of IgAV (15, 16). However, the pathogenic mechanisms of IgAV are far from being completely understood. Immunological factors other than Gd-IgA1-containing immune complexes, such as anti-endothelial cell antibodies (AECAs), and irritant causes of disease may also be important. There is also the issue of the exploration of mechanisms that lead to different clinical phenotypes. Furthermore, it is still inconclusive whether IgAV is an extension of IgAN or a different disease entity. The exploration of the applicability of the IgAN pathogenesis hypothesis to IgAV can deepen the understanding of the disease and enhance the standardized diagnosis and management, and the current molecular mechanism-based targeted therapy for IgAN may be able to be applied to IgAVN. This paper will describe the epidemiological characteristics of IgAV, and review the advances in IgAV pathogenesis in terms of Gd-IgA1, complement system, cellular immunity, and other immunological factors based on the “multiple-hit” models, as well as environmental and genetic factors, and summarize the main biomarkers of IgAV, in order to shed light on guiding the prevention and management of the disease.

## 2 Epidemiology

### 2.1 Overview of incidence

The incidence of IgAV has been relatively stable over time, generally showing an upward trend. The change in the incidence of IgAV may be correlated with the updated inclusion criteria for the epidemiological study population. The diagnosis criteria applied to IgAV earlier is the 1990 American College of Rheumatology (ACR) classification criteria, which is not specific for children, showing a restricted applicability. In 2010, the European League against Rheumatism/Pediatric Rheumatology International Trials Organization/Pediatric Rheumatology European Society (EULAR/PRINTO/PRES) published the diagnostic criteria for children: purpura or petechiae (mandatory) and meeting at least one of the following criteria: abdominal pain, arthritis or arthralgia, renal involvement, and biopsy of IgA deposition at any site (sensitivity 100%, specificity 87%), which is still the accepted classification standard (17). A French study in 2017 applied the EULAR/PRINTO/PRES criteria to homogenize the study population for the first time, finding that the average annual incidence rate was approximately 30 per 100,000 people with capture-recapture analysis (18). There are differences in the incidence rate of IgAV among countries; e.g., the average annual incidence is 6.79/100,000 in Croatia, 6.21–20.4/100,000 in the UK, 6.1/100,000 in the Netherlands, 17.55/100,000 in southern Sweden, 12.9/100,000 in Taiwan of China, and 55.9/100,000 in Korea (19–21). The actual incidence might be higher, considering that children with isolated cutaneous IgAV generally require no hospitalization, which results in underestimated epidemiological data fetched from healthcare facilities; furthermore, the chronic disease management and follow-up systems in some regions are imperfect and there may be missing data due to incomplete registration. There are no data yet referring to systemic or skin-limited vasculitis alone since this distinction has not been made until recently.

### 2.2 Region and race

The variation in incidence from country to country is described above. Modern geostatistical studies in Croatia have shown that IgAV is geospatially aggregated (22), appearing as a non-random distribution and mainly clustered around the Mediterranean and the western continental regions. In addition, there are ethnic disparities in IgAV; the incidence is 3–4 times as high in Caucasian or Asian children than in black children (23).

### 2.3 Age and gender

IgAV can occur at any age, mainly in childhood, the estimated incidence rates among children are 2 to 33 times greater than those in adults (9), 75%–90% of pediatric patients are below 10 years old, and the most frequent being 4–7 years of age, with an incidence up to 70.3/100,000 (23). The age-preference may be attributed to the fact that children of this age group are a favored population for pathogenic infections. When comparing genders, males seem to be more susceptible than females (23) and the prevalence ratio of males to females is approximately 1.5:1. However, the directivity of this slight gender difference is not yet clear.

### 2.4 Seasonal variation

The peak onset of IgAV appears in the fall-winter season, while summer shows the lowest rates of attack (18), which is consistent with epidemics of most respiratory infections. The temporal pattern of IgAV attack provides a clue to trace the association between infection and the pathogenesis of IgAV. Moreover, we have observed a decrease in the frequency of IgAV during the coronavirus disease 2019 (COVID-19) pandemic. This reduction could be associated with the precautions (such as wearing masks and quarantining) and decreased circulation of respiratory viruses (24).

## 3 Pathogenesis

**Figure 1:**
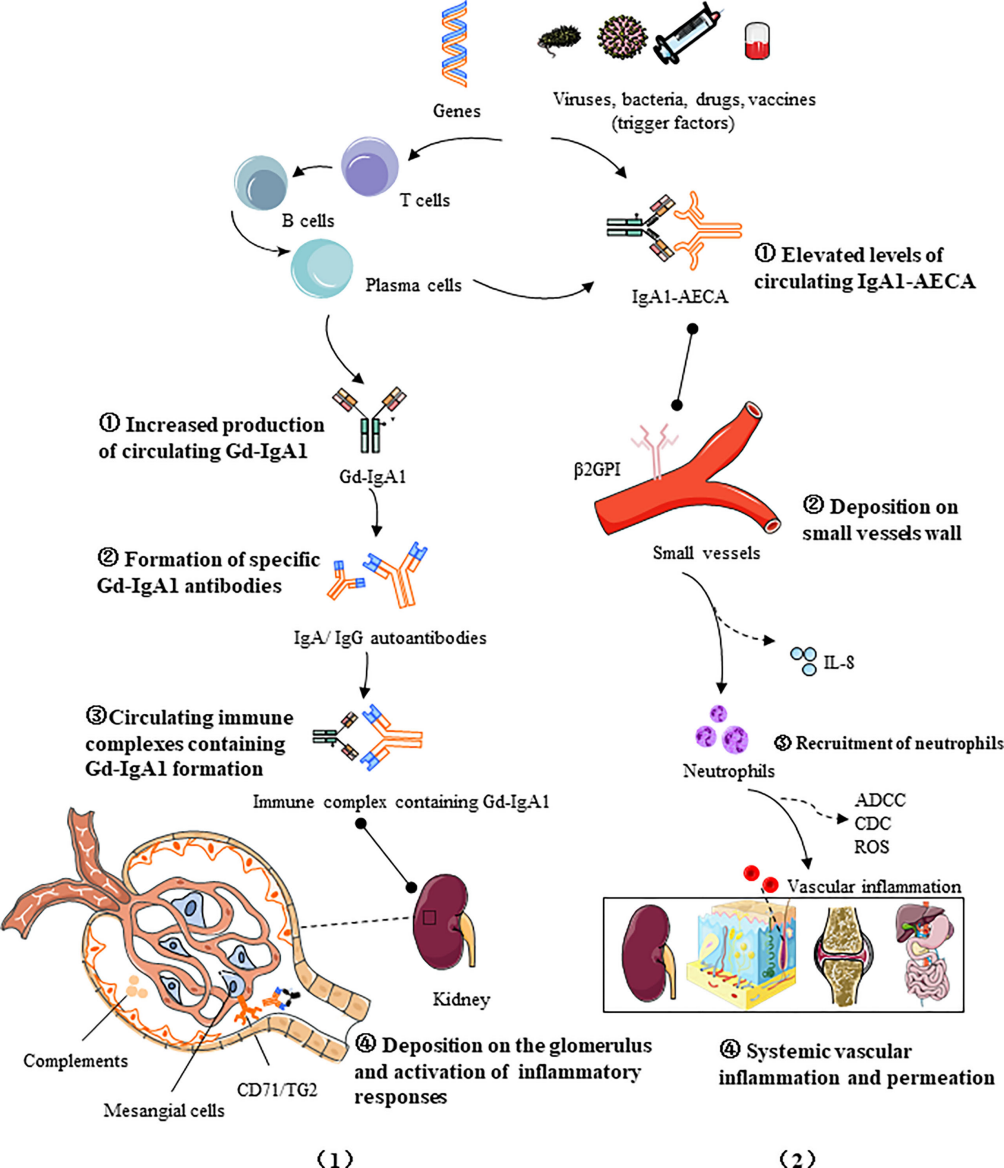
Two four-hit pathogenesis models for IgAV: (1) Increased production of circulating galactose-deficient IgA1 (hit1) binds to specific IgA1 autoantibodies (hit2), forming pathogenic circulating immune complexes (hit3), which then deposited in the glomerulus and trigger inflammatory responses (hit4); (2) The first hit is provided by elevated AECA levels of IgA1 isotype, followed by the binding of IgA1-AECA complexes to specific β2 glycoprotein I receptors (β2GPI) on vascular endothelial cells (hit2), inducing excessive production of proinflammatory factors such as IL-8, which in turn stimulates neutrophil recruitment (hit3), and then the activation of causes extensive damage to the vascular endothelium *via* antibody-dependent cellular cytotoxicity (ADCC), complement-mediated cytotoxicity (CDC), and reactive oxygen species (ROS), ultimately leading to systemic vascular inflammation and permeation (hit 4). The pathogenic processes of IgAV are promoted by immune cells and inflammatory mediators and regulated by multiple factors, including genetics and the environment.

IgAV is a leukocytoclastic vasculitis with an IgA-dominant immune complex deposited within or around the small vessels. Two major models of pathogenesis for IgAV have been proposed to interpret the nephritis and systemic phenotypes, respectively. IgAVN manifests similarly to IgAN, both characterized by hematuria, proteinuria and glomerular mesangial immune complex deposition, and its pathogenesis can be explained by a similar “four-hit” theory (25), that is, increased production of circulating galactose-deficient IgA1 (hit1) binds to specific IgA1 autoantibodies (hit2), forming pathogenic circulating immune complexes (CICs) (hit3), which then deposits in the glomerulus and triggers inflammatory responses (hit4).This hypothesis highlights the critical role of Gd-IgA1 in renal injury. However, some publications stated that in a certain number of patients there is no obvious increase of Gd-IgA1 in serum or in biopsy specimens, while in a certain number there is an increase in Gd-IgA1 and no clinical manifestations of the disease (26, 27). In contrast, another hypothesis targets the systemic manifestations of IgAV, emphasizing the effect of neutrophil activation on systemic small vessels (25, 28, 29). The first hit is provided by elevated AECA levels of IgA1 isotype (30), followed by the binding of IgA1-AECA complexes to specific β2 glycoprotein I receptors on vascular endothelial cells (hit2), inducing excessive production of proinflammatory factors such as interleukin-8 (IL-8), which in turn stimulates neutrophil recruitment (hit3), and then neutrophils are activated by the interaction of IgA1 and IgA1 Fc alpha receptor I (FcαRI, also called CD89), causing extensive damage to the vascular endothelium wall *via* antibody-dependent cellular cytotoxicity (ADCC), complement-mediated cytotoxicity (CDC), and reactive oxygen species (ROS), ultimately leading to systemic vascular inflammation and permeation (hit4). It is to be noted that there are few original articles on the role of AECA in IgA vasculitis, and it is possible that AECA is an incidental phenomenon of vascular injury and cannot be completely ruled out. The pathogenic processes of IgAV are promoted by immune cells and inflammatory mediators and regulated by multiple factors, including environmental and genetics (**Figure 1**).

### 3.1 Galactose-deficient IgA1

#### 3.1.1 The production of galactose-deficient IgA1

IgA is the most abundant immunoglobulin class found in mucosal immunity, produced by B cells through class switching in a T-cell-dependent or T-cell-independent manner (31). It is mainly generated in mucosal-associated lymphoid tissue and bone marrow. According to the structure of the hinge region, IgA can be divided into IgA1 and IgA2. IgA1 makes up to 90% of the serum IgA (32). IgA1 exists in both monomeric and polymeric forms (composed of two to four monomers linked through joining chains). More than 90% of serum IgA1 is monomeric, while the type of IgA1 secreted by mucosal tissues is mainly polymeric. Compared with IgA2, IgA1 is unique in that it is rich in O-linked glycosylation structures, for which two octapeptide repeat regions are inserted between the C1 and C2 regions of its heavy chain, with three to six O-glycosylation sites consisting of serine or threonine residues (30). Under the catalysis of polypeptide N-acetylgalactosaminyl transferase 2 (GALNT2), it can combine with N-acetylgalactosamine (GalNac) to form O-glycans (33), and the terminus of which is bound to galactose or sialic acid by the action of core-1 β1,3galactosyltransferase (C1GALT1, encoded by the *C1GALT1* gene) and acetylgalactosamine-specific α-2,6 sialic acid transferase (ST6GALNAC2, encoded by the *ST6GALNac2* gene) respectively (34, 35), completing the glycosylation process. The correct folding and complete activity of C1GALT1 requires the participation of a specific chaperone, *COSMC* (encoded by the *C1GALT1C1* gene) (36). Altered expression of activity key transferase during the glycosylation steps, such as low expression of *C1GALT1* and *COSMC*, and upregulation of *ST6GalNac2* can hinder the IgA1 glycosylation process (37, 38), resulting in the exposure of terminal GalNac residues or the production of sialized Gd-IgA.

#### 3.1.2 Regulatory mechanisms of galactose-deficient IgA1

The enzymatic activity and gene expression in the process of glycosylation are regulated by certain cytokines and related molecular signaling pathways (39). It is currently considered that Th2 cytokines, especially the interleukin-6 (IL-6) family, are most closely related to Gd-IgA1 (40). IL-6 is the main candidate factor for promoting the terminal differentiation and the proliferation of IgA1 (39). Aberrant IL-6 activates the JAK2/STAT3 signaling pathway through its receptor protein coupling gp130, and the enhanced phosphorylation of STAT3 further downregulates the transcription of *C1GALT1*, thereby mediating the production of Gd-IgA1 (41). Furthermore, another member of the IL-6 family, leukemia inhibitory factor (LIF), upon receptor binding, leads to the overproduction of Gd-IgA1 *via* the LIF/STAT1 pathway (42). In addition to the IL-6 family, B-cell activating factor (BAFF), a tumor necrosis factor (TNF) superfamily member, is also involved in the synthesis of Gd-IgA1. BAFF stimulates B-cell transformation (43). It blocks the degradation of NF-kB and leads to enhanced translocation to the nucleus through the binding to BAFF receptors, such as B-cell maturation antigen (BCMA), the transmembrane activator and cyclophilin ligand interactor (TACI). The activation of NF-kB signaling subsequently impacts on the production of Gd-IgA1 (44). Notably, a proliferation-inducing ligand (APRIL), also known as tumor necrosis factor ligand superfamily member 13 (TNFSF13), shares the same signaling receptor as BAFF, also playing an important role in IgA class transformation and plasmacytoid differentiation (44, 45), is similarly involved in the formation of aberrant glycosylated IgA (46).

Recent studies have found that some mucosal immunity-related molecule Toll-like receptors (TLRs), also participate in the production of Gd-IgA1 (47). For example, the activation of TLR4 causes the methylation of *C1GALT*, leading to reduced *COSMC* expression and a low glycosylation level of IgA1; TLR7 can stimulate B cells to secrete cytokines such as IL-6 and IL-12 and generate more Gd-IgA1 through the TLR7–GALNT2 axis (48); TLR9 activation can induce increased IL-6 or APRIL production through the TLR9–MyD88 signaling pathway as well as promote Gd-IgA1 in synergy with both (49).

**Figure 2:**
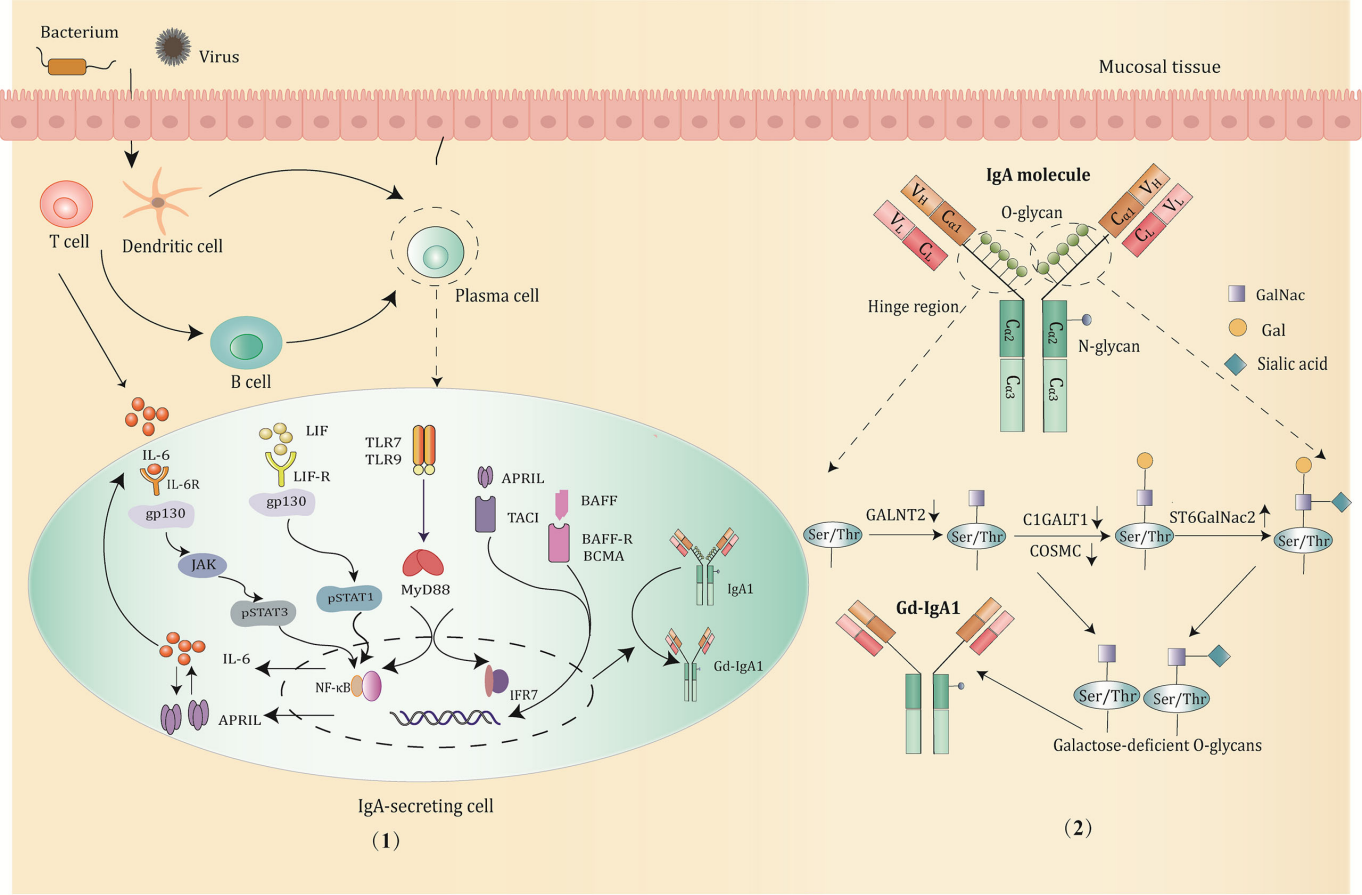
The production and major regulatory mechanisms of Gd-IgA1. (1) B cells in the mucosa differentiate into IgA1-secreting plasma cells and produce IgA1 through class switching in a T-cell-dependent or T-cell-independent manner when stimulated by pathogens, such as bacterium and virus. Then, the abnormal activation of signaling pathways promotes the production of Gd-IgA1 through altering the expression and activity of the key glycosyltransferases. Potential signaling pathways involved are mainly below: IL-6 activates the STAT3 pathway *via* binding to its gp130 receptor; LIF binds the LIF-R/gp130 receptor and activates the STAT1 pathway; TLRs (TLR7/TLR9) activation induce production of IL-6 and APRIL, which function synergistically to promote the generation of Gd-IgA1; BAFF or APRIL signaling through the BAFF receptors, such as TACI, BAFF-R, BCMA prevents degradation of NF-κB and influence the O-glycosylation of IgA1. (2) The structure and O-glycosylation process of IgA1. IgA1 has O-glycans located in the unique hinge region, with 3 to 6 O-glycosylation sites consisting of serine or threonine residues. The low expression of GALNT2, C1GALT1, and COSMC, and upregulation of ST6GalNac2 can hinder the IgA1 glycosylation process, resulting in the exposure of terminal GalNac residues and overproduction of Gd-IgA1.

In addition to the aforementioned molecules, the expression of glycosyltransferase can also be regulated by certain miRNAs. For example, *C1GALT1* expression is negatively correlated with miR-148b. The upregulation of miR-148b leads to an increase in the Gd-IgA1 level due to suppressed *C1GALT1* expression (49). It is worth noting that most of the studies on the production and regulatory mechanism of Gd-IgA1 are based on IgAN patients, and exploring whether there are similar molecular signaling pathways in IgAV can provide novel targets for therapy (**Figure 2**).

#### 3.1.3 Correlation between Gd-IgA1 level and the disease

Gd-IgA1 plays an important role in the pathogenesis of IgAV. Some studies showed that the levels of serum IgA1 as well as Gd-IgA1 are significantly increased in IgAVN, with the acute phase being higher than in the remission (50–54). At present, the approaches used to detect Gd-IgA1 mainly include the conventional *Helix aspersa agglutinin* lectin enzyme-linked immunosorbent assay (ELISA) method and the novel lectin-independent ELISA assay using a specific monoclonal antibody *KM55* (55, 56). Suzuki et al. found that Gd-IgA1 mesangial deposition was a typical manifestation of IgAN and IgAVN patients when they performed immunohistochemical analysis of renal biopsy specimens from those and patients with other secondary nephropathy by *KM55* staining (57), which has also been confirmed in relevant studies on children (58). Gd-IgA1 levels in patients of IgAV with nephritis were similar to IgAN, while those without nephritis showed no statistical difference compared to healthy controls (58). Neufeld et al. also showed that although Gd-IgA1 was deposited around the blood vessels in both the skin-limited and systemic types, the serum Gd-IgA1 level was significantly higher in the latter (27). This suggests that there may be a dose-dependent effect on the pathogenicity of Gd-IgA1, and IgAVN can only occur when Gd-IgA1 accumulates to a certain level. However, the Gd-IgA1 threshold for different clinical phenotypes of IgAV needs to be further explored. Studies related to the predictivity of Gd-IgA1 on disease severity have shown that the level of Gd-IgA1 does not correlate with the degree of renal involvement; its level can only signal whether the kidney is involved but cannot predict the extent of proteinuria, GFR, and renal pathology score (55). In clinical practice, anti-CD20 therapy with rituximab, which depletes B cells and reduces the production of Gd-IgA1, has been used with benefit in cases of IgAV or IgAVN refractory to conventional immunosuppressive drugs (59). Simultaneously, a novel targeted-release formulation of budesonide, which can target Payer’s patches in the ileum and suppress the gastrointestinal immune system and subsequently decrease the level of Gd-IgA1, could become a promising treatment for IgAV (60).

### 3.2 Circulating immune complexes containing Gd-IgA1

#### 3.2.1 Autoantibodies

High serum Gd-IgA1 levels are predisposed to familial distribution since circulating galactose-deficient IgA1 has also been found in first-degree relatives of many patients with IgAV and IgAN, yet these relatives often show no clinical evidence of renal disease (61). In addition, in 2020, Ishiko et al. found that patients with lupus nephritis and primary membranous nephropathy also had renal Gd-IgA1 deposition (58). These suggest that Gd-IgA1 alone is not a determinant of IgAV pathogenesis and that other triggers need to be considered. IgAVN patients are similar to IgAN patients in the presence of IgG autoantibodies against Gd-IgA1 in the serum, and the level is higher in the acute period of nephritis than in the resolution phase (37), and co-sedimentation of IgG and Gd-IgA1 immune complexes is seen in the kidney mesangial region by confocal microscopic analysis (25). In studies of IgAN, specific IgG autoantibodies were found to correlate with disease activity as well as renal prognosis (62). Berthoux et al. found that mean serum levels of total autoantigen, normalized IgG autoantibody, and total IgA autoantibody were significantly higher in 97 adult patients with IgAN than in the healthy controls or patients with non-IgAN disease, furthermore, IgG autoantibody levels ≥1.33 predicted dialysis or death in Cox regression and Kaplan–Meier analyses (63). In addition, another study revealed a correlation between the concentrations of the autoantigen and the corresponding IgG autoantibodies in serum samples from 135 patients with biopsy-proven IgA nephropathy, 76 patients with other renal diseases, and 106 healthy controls. Serum IgG autoantibody and Gd-IgA1 can influence each other (64). However, the correlation of autoantibodies with disease activity and severity in patients with IgAV needs further confirmation.

#### 3.2.2 Gd-IgA1 immune complex formation

In fact, the formation of the Gd-IgA1 circulating immune complex (CIC) is divided into two major parts: Firstly, abnormalities in the quantity and structure of IgA alter the binding affinity to its receptors, which include the transmembrane receptor on the surface of circulating myeloid leukocytes—FcαRI (CD89), and the transferrin receptor (also referred to as CD71) on mesangial cells. It was shown that Gd-IgA1 forms the IgA1–CD89 complex with soluble FcαRI (65, 66); Secondly, the exposed GalNac residues at the end of Gd-IgA1 serve as new binding epitopes to the corresponding anti-Gd-IgA1 autoantibodies (including IgA and IgG) in circulation, leading to the generation of Gd-IgA1-autoantibody-CD89 CICs (67, 68). CICs can further cause the aggregation of IgA, forming polymers of larger molecular weights.

The weights and fractions of CICs appear to influence the disease phenotype. As early as 1979, a study by Levinsky and Barratt found that IgAV patients all had relatively small IgA1-circulating immune complexes, while those with nephritis contained additional large-molecule IgA1–IgG immune complexes (69). Later, this phenomenon has been verified by other scholars (70), who found that CIC molecules are usually >800 ku and contain polymerized IgA and IgG in IgAVN patients (71). Macromolecular immune complexes containing Gd-IgA1 cannot be effectively recognized by the asialoglycoprotein receptor (ASGPR) on hepatocytes (72). The impaired hepatic clearance may be a reason for enhanced levels of CICs. CICs enter the kidney *via* the blood flow, and the binding of CD71 on mesangial cells leads to deposition within the glomerulus, which is synergistically promoted by other molecules secreted by mesangial cells such as extracellular matrix proteins, integrins, and transglutaminase 2 (TG2) (73–75). The induction of TG2 in turn up-regulates CD71 expression, showing a positive feedback enhancement effect of CICs on renal deposition (76). The deposition sites of immune complex IgAVN differ from those of IgAN in that IgAVN exhibits mesangial, subendothelial, and perivascular deposits, while the CICs of IgAN are mainly deposited in the mesangial region (3, 77).

### 3.3 Renal pathology and local immune microenvironment

#### 3.3.1 Renal pathology

IgAVN is defined as mesangial proliferative glomerulonephritis and immunofluorescence indicates that the sediments are predominantly IgA1, often with C3 and occasionally with IgG and IgM, with granular or diffuse subcellular distribution in the mesangial area or the capillary wall (78). The histological lesions of IgAVN range from mild to severe, manifesting as slight mesangial proliferation, microscopic lesions, and focal nephritis in mild types, and diffuse proliferative nephritis, segmental glomerulosclerosis, or crescent formation in severe cases (79). It remains controversial and unresolved which histological classification to choose in regard to the interpretation of renal biopsy findings. Compared with IgAN, the episode of kidney injury in IgAVN is mainly acute, usually accompanied by intracapillary proliferation and inflammatory cell infiltration, with more crescentic lesions and fewer sclerotic lesions than IgAN (80, 81). Therefore, the most widely used classification for IgAVN is still the International Study of Kidney Disease in Children (ISKDC), based on crescents to a large extent, which focuses on active inflammation while ignoring vascular and tubulointerstitial changes. It was subsequently recognized that sclerotic glomeruli and interstitial fibrosis correlate better with the long-term outcome. Therefore, the Oxford classification is increasingly used. The revised 2016 Oxford classification includes five main parameters: mesangial hypercellularity (M), endocapillary proliferation (E), segmental glomerulosclerosis/adhesion (S), tubular atrophy/interstitial fibrosis (T), and cellular/fibrocellular crescents (C), and current studies have suggested that T and S lesions are associated strongly with renal progression, however, a larger multicenter study with a longer follow-up targeted at IgAVN patients is needed to validate the predictive value of MEST-C in IgAV (82–84).

IgAVN is caused by a cascade of immunological reactions triggered by circulating Gd-IgA1 immune complexes, which are deposited in the glomerular mesangium and small vessel walls through the synergistic effects of transferrin receptor and TG2 on mesangial cells. The deposits of CIC further stimulate mesangial cell proliferation and extracellular matrix expansion, inducing the aggregation of inflammatory cells such as neutrophils and T lymphocytes and activating the complement system. Mesangial cells or inflammatory cells that migrate to the glomerulus can produce excessive cytokines and simultaneously release multiple pro-inflammatory factors, such as prostaglandins, angiotensin II, nitric oxide synthase, chemokines, matrix metalloproteinases (MMPs) and cytokines or key mediators affecting podocyte functions, ultimately leading to renal damage (85–87). In addition, there is a tubular-glomerular feedback mechanism that further exacerbates glomerular and tubulointerstitial inflammation, resulting in a series of clinical consequences such as proteinuria, hematuria, renal insufficiency, and hypertension.

#### 3.3.2 Local immune microenvironment

##### 3.3.2.1 Complement activation

The depositions of complement components such as complement factor B, C3, C4d, and C5b-9 complexes are often observed in skin and kidney biopsy specimens from IgAV and IgAVN patients during the acute stage, whereas serum complement levels are lowered (88, 89). The immune complex containing Gd-IgA1 activates the complement cascade *via* the mannose-binding lectin pathway and the alternative pathway once deposited in subendothelial and mesangial areas (90–92), in which the lectic pathway is dominant (93), and the supporting evidence is mainly listed below: At present, glomerular deposition of the lectin pathway-related molecules mannose binding lectin (MBL) and mannan-binding lectin serine peptidase (MASP) and an elevated plasma C4d level have been detected (93); a case report has shown that IgAV patients presenting with acute progressive nephritis who were treated with anti-MASP-2 monoclonal antibody *Narsoplimab* had a sustained reduction in lectin pathway activity and a delayed progression of renal dysfunction (94); IgAVN and IgAN patients with C4d deposition had a higher incidence of chronic lesions, low GFR, and poor prognosis, and were more likely to develop microangiopathy (95–97).

Complement activation can lead to fibrin deposition, glomerular basement membrane disruption, attraction of inflammatory cells, endothelial cell activation, and increased secretion of proinflammatory cytokines. Meanwhile, cytokines can induce the proliferation of epithelial cells in Bowman’s capsule and eventually the mesenchymal fibroblast infiltration, resulting in the formation of fibrous crescents (3). The complement cascade is strongly associated with the renal pathology and clinical phenotypes in IgAV patients, and biologics directed toward the complement pathway may be a new strategy for the treatment of IgAV in the future (98).

##### 3.3.2.2 T lymphocyte-mediated immune imbalance

In addition to attacking renal resident cells, Gd-IgA1 circulating immune complexes also affect T lymphocyte immunity, manifesting as altered quantity, proportion, and function of T lymphocytes. Imai et al. found that cytotoxic T cells (CTLs) were activated in IgAVN patients with nephrotic-range proteinuria, the expression of *GNLY* and *GZMB* as well as the level of granulysin and granzyme B in peripheral blood were increased, and the glomerular CTLs behaved similarly (99); Furthermore, it has been described that the expression of CX3CR1 on the surface of CTLs is up-regulated, in line with increased serum and glomerular CX3CL1 expression, and granulysin and granzyme B facilitate the transmigration of CTLs through blood vessels to the glomerulus (100), resulting in increased vascular permeability and accelerated cell death. Helper T cells (Th cells) can be classified into different subsets: Th1, Th2, Th17, follicular helper T (TFH), and regulatory T (Treg) cells, which also serve central roles in IgAV. Multiple studies have demonstrated that a Th1/Th2 imbalance exists in both the blood and renal tissues of IgAVN patients in the acute phase (101). The intranuclear expression of *GATA3*, a hallmark transcription factor of Th2 cells, was significantly increased in IgAVN, while there was no obvious change in the level of Th1-specific transcription factor *Tbet*, showing a state of Th2 dominance. IgAV patients also have an imbalance in the Th17/Treg and present with increased Th17 cells and decreased Treg cells (102). Recent studies have identified a special type of Treg cell, Tr1 cells, co-expressing *CD49b* and *lymphocyte activation gene 3* (*Lag-3*). Tr1 cells can be used as a risk factor for predicting the relapse of IgAV, with increased expression in renal tissues and significantly decreased percentage in peripheral blood during the acute phase, and partial recovered once the disease remitted, those who with low Tr1 cells during remission exhibited a high recurrence rate (103).

Furthermore, IgAV is accompanied by increased Tfh cell frequencies in acute stage, the elevation degrees vary from subpopulations, and reduced amount of Tfh cells in the remission (104). There are different correlations with organ-specific clinical manifestations among each subtype of Tfh cells (105).

In summary, most IgAV patients show an increase in the number of Th2, Th17, and Tfh cells with a concomitant decrease in the population of Treg cells. Abnormal T lymphocyte subsets can cause corresponding changes in cytokines, serum levels of IL-6, IL-8, IL-17A, IL-18, IL-23, and TNF-α in IgAV patients are significantly higher than that in healthy controls, while the negative regulators IL-10, IL-27, and TGF-β1 are obviously lower (103, 106, 107). For instance, serum proinflammatory cytokines can stimulate the production of chemokines and adhesion molecules by endothelial cells and attract other inflammatory cells, driving inflammatory responses. For instance, IL-6 has been shown to prime polymorphonuclear neutrophils *in vitro* and induce NETosis (108, 109). At the same time, cytokines affect the IgA glycosylation mechanism through relevant molecular signaling pathways, which intensify the production of Gd-IgA1. The formation of a positive feedback loop between inflammatory mediators and immune cells exacerbates kidney damage in IgAV.

### 3.4 IgA1-anti-endothelial cell antibodies and systemic small-vessel inflammation

The typical histopathological findings of IgAV are endothelial cell injury and infiltration of leukocytes within the vascular wall. Since the action of circulating immune complexes containing Gd-IgA1 cannot fully explain systemic symptoms in IgAV, and the “new multiple hit” hypothesis provides supplementary explanations of IgAV pathogenesis, we propose that the binding of IgA1 to anti-endothelial cell antibodies (AECAs) plays a central role in systemic small-vessel inflammation. AECAs are a heterogeneous group of autoantibodies bound to endothelial cells (ECs) through region-specific interactions and target a variety of antigens, both against EC antigens and antigens adsorbed on EC membranes. AECAs are commonly of the IgG isotype, and a few are IgM and IgA types. They have been described in a variety of vascular diseases, such as Takayasu arteritis, giant cell arteritis, Kawasaki disease, and other systemic vasculitis (110, 111). In 1998, Fujieda et al. found the presence of IgA-anti-endothelial cell antibodies in IgAV, and IgA-AECAs were able to bind to bovine glomerular ECs (29). Recently, Yang et al. also detected IgA in AECAs of human dermal capillary endothelial cells and human umbilical vein endothelial cells from the serum of children with IgAV, but IgM and IgG failed to be detected, supporting that it is mainly the IgA isotype that plays a role in IgAV (28). IgA-AECAs may be produced by the molecular mimicry during microbial infection, and high IgA-AECAs titers correlated with active disease, while antibody titers fall in remission (112). Tumor necrosis factor (TNF-α) can significantly increase IgA-AECA levels by mediating the exposure of EC-specific antigenic epitopes. IgA-AECAs bind to specific β2GPI receptors that adhere to endothelial cells (113), inducing the release of IL-8 *via* the MEK/ERK signaling pathway (114), and therefore recruiting polymorphonuclear neutrophils *in vitro*. Meanwhile, IgA contributes to neutrophil activation and chemotaxis through its binding to FcαRI receptors, and the cross-linking of FcαRI causes endothelial cell injury in multiple ways, such as phagocytosis, ROS generation, release of particles containing toxic molecules such as lactoferrin, secretion of cytokines and chemokines, antibody-dependent cellular cytotoxicity, and the formation of neutrophil extracellular traps (NETs) (115). In addition, serum IL-33 level was found to be positively correlated with AECA-IgA concentration (116), and elevated serum IL-33 may be a result of endothelial cell injury, which in turn interact with endothelial cells as a positive feedback, promoting the production of adhesion molecules, endothelial selectin and monocyte chemotactic protein-1 (MCP-1) and bringing neutrophils and endothelial cells into close contact (117), further aggravating vascular endothelial dysfunction and amplifying immune inflammatory response.

### 3.5 Irritant causes of disease

#### 3.5.1 Infection

Many studies have supported the correlation between infection and the onset of IgAV, and the evidence can be described as follows: The prevalence of IgAV is seasonal, consistent with the epidemiological characteristics of some respiratory or enteropathogenic microorganism (118); IgAV recurrence is associated with reinfection of certain pathogenic microorganisms, for that IgAV patients with *Helicobacter pylori* (*Hp*) infection could achieve remission *via Hp* eradication, but relapsed after re-infection with *Hp* (119, 120).

Approximately 70% of IgAV patients have a history of prodromal infection or co-infection symptoms at the disease onset, with respiratory tract infections being the most common, followed by gastrointestinal infections, cellulitis, and urinary tract infections, etc. The common infectious pathogens are *Streptococci*, *Hp*, *Mycoplasma pneumoniae*, *Human microvirus B19*, and *Hepatitis A virus* (121, 122). IgA is synthesized by plasma cells in mucosa-associated lymphoid tissue (MALT), which includes the nasopharynx, tonsils, and gastrointestinal mucosa (such as Peyer’s patches and mesenteric lymph nodes). When the body is stimulated by pathogens, B cells differentiate into mature plasma cells, leading to the secretion of more IgA polymers (123, 124). In turn, pathogens may contain antigenic structures similar to those of the vessel walls that induce cross-reactive autoantibodies and enhance immune responses (28). Additionally, infections up-regulate pro-inflammatory factors such as IL-6 or pattern recognition receptors, leading to changes in the key enzyme activities of IgA1 glycosylation. As an example, TLR9 expression was discovered to be increased in tonsillitis, resulting in activation of APRIL and thus causing the overproduction of Gd-IgA1.

It should be pointed out that some studies support that SARS-CoV-2 has been linked to IgAV. The mechanisms involved in SARS-CoV-2 regulation of autoantibody generation, faulty development of Th2 response, endothelial inflammation and dysfunction, complement activation, NET production, and the upregulation of proinflammatory cytokines such as interleukin 6 may lead to vasculitis (125–127). The contradiction between the reduction in IgAV cases and the possible trigger may arise from the fact that SARS-CoV-2 may not be as strong a trigger as the other cold viruses or bacteria for IgAV.

#### 3.5.2 Non-infectious triggers

Besides infection, drugs also contributed to the development of IgAV. A case-crossover control study by Piram et al. in 2018 concluded that vaccination with common vaccines in childhood (*DPT*, *polio vaccine*, *meningococcal vaccine*, and *hepatitis vaccine*) did not significantly increase the risk of IgAV within 3 months (128). However, a French study combined with the *French Pharmacovigilance Database* (FPVD) and the World Health Organization (*WHO*) Global Individual Case Safety Report (ICSR) database revisited the relations between drugs and IgAV using a dual pharmacovigilance-based approach in 2021, which was inconsistent with previous reports, this study believed that vaccines are the primary suspected IgAV-inducing drugs, mainly for *influenza* and *measles vaccines*, others being *rubella*, *mumps*, *polio*, *diphtheria*, *tetanus*, and etc. (129). In the context of the COVID-19 pandemic, some scholars suggest a link between the increase in anti-SARS-CoV-2 spike IgA and the reactivation of pre-existing IgA vasculitis observed after COVID-19 vaccination (130). Vaccines may act as an immunological trigger by mimicking the pathogen-specific immune response and inducing long-term antibody production, promoting the development of IgAV. The second most relevant drugs are antibiotics (such as β-lactams, quinolones, and macrolides, which encompass almost all pharmacological classes of antibiotics), the role of which is controversial given that infection may be a confounding factor in the analysis of antibiotic effects. Further, biological agents, especially TNF-α blockers (*adalimumab*, *infliximab*, etc.) are also commonly suspicious drugs, and case reports described the occurrence of IgAV with rare neurological involvement in Crohn’s patients after adalimumab treatment (131). Studies have suggested that TNF-α blockers can stimulate the formation of anti-tumor necrosis factor or tumor necrosis factor-containing immune complexes in small vessels and subsequently activating the complement pathway to induce vasculitis (132). Other drugs that evoke IgAV include rosuvastatin, tofacitinib, and so on (133, 134). Altogether, drugs may induce IgAV through stimulation of antibody production, direct toxic effect on the blood vessel wall, or activation of eosinophils.

### 3.6 Genetic predisposition

The prevalence of IgAV differs with geographical variation and ethnicity, and it has been reported that some patients present with familial aggregation (135). Moreover, the clinical course, manifestations, and prognosis vary highly between individuals, underscoring the importance of genetic factors in IgAV pathogenesis. The susceptibility of IgAV is not determined by a single gene, which requires a combination of genes to work together. Current research mainly focuses on gene polymorphisms related to signaling pathways associated with immune response and inflammation, involving the human leukocyte antigen (HLA), cytokines and their receptors, the complement system, endothelial function, coagulation, and fibrinolytic systems. At present, there are various methods for studying genetic elements, such as whole-genome sequencing and copy number variation analysis, rearrangement of the genetic structure (small insertions or deletions, segmental duplications, gene expression profiling, etc.). However, the majority of studies only focused on one gene or a few genes. Larger-scale and more systematic studies are needed to verify the effect of gene polymorphisms on IgAV. Investigating genetic risk factors is helpful for deepening the understanding of IgAV pathogenesis, and close monitoring and prompt intervention of patients carrying susceptible genotypes can help to avoid the occurrence of serious complications, improve disease prognosis, and achieve individualized precision therapy.

#### 3.6.1 HLA

Human leukocyte antigens, also known as major histocompatibility complex (MHC) antigens, are vital components of the immune system, the hyper-polymorphic genetic loci of which are situated on the short arm of chromosome 6 (6p21). MHC genes are mainly divided into three categories: MHC class I (including HLA-A, HLA-B, and HLA-C), MHC class II (comprising of HLA-DR, HLA-DP, and HLA-DQ) and MHC class III genes [encompassing genes encoding complement components, heat shock protein 70 (HSP70), tumor necrosis factor and receptor for advanced glycosylation end products (RAGE; also known as AGE)] (136). Among them, MHC class II genes have been identified as the most predominant genetic susceptibility loci for IgAV.

In 2017, the first genome-wide association study (GWAS) for IgAV in Spain found that polymorphic linkage disequilibrium blocks located in the intergenic regions of HLA-DQA1 and HLA-DQB1 are closely related to disease susceptibility, with the strongest association signal being HLA-DRB1 alleles, particularly HLA-DRB1 loci 11 and 13 (137) (98). The effects of different genotypes on IgAV are not identical. For instance, HLA-DRB1*03 and HLA-DRB1*07 have protective effects against the development of IgAV, while HLA-DRB1*01 and HLA-DRB1*11 allele groups may predispose individuals to IgAV (138–140). Notably, population disparities exist in the effect of HLA-DRB1 polymorphism, e.g., studies in Caucasians have shown that HLA-DRB1*01 increases susceptibility to IgAV (138), whereas this result has not been validated in India (141). HLA class II gene polymorphisms can not only confer susceptibility to IgAV but also influence disease severity and clinical heterogeneity.

In addition to the HLA-class II region, the other two classes are also known to affect the susceptibility of IgAV. A study in Turkey found an increased frequency of HLA-class I alleles coexistence in IgAV and HLA A2, A11, and B35 could increase the risk of IgAV, while those carrying HLA A1, B49, and B50 antigens had a reduced disease risk (142), and further analysis revealed that HLA B35 was associated with renal involvement and HLA A3 and B44 were more susceptible to exhibiting joint symptoms. There are few studies on the HLA-III class region. The sites in this region are primarily associated with the synthesis of complement and glycosylation-related enzymes of IgA. Activation of complement 4 is a key molecule in the complement cascade, whose gene expression loss leads to the reduced synthesis (143), and individuals with C4 gene homozygous deletion are more prone to IgAVN (144).

#### 3.6.2 Non-HLA

Recently, a variety of non-HLA genes have been found to be involved in IgAV susceptibility. Carmona et al. identified a shared potential risk locus between IgAV and KD in a large-scale crossover disease meta-analysis, rs3743841 (located in the intron region of the *NAGPA* gene), which is the strongest non-HLA signal with IgAV to date (145). This study revealed that *NAGPA* regulates the expression level of *NAGPA* proteins and participates in the immunomodulatory response of the body by acting on the central link of the lysosomal pathway. This study also revealed that the lysosomal pathway may play an important role in the pathogenesis of IgAV. Most studies suggest that patients with familial Mediterranean fever (FMF, the disorder caused by *MEFV* gene mutations) are at an increased risk of IgAV (146, 147). *MEFV* encodes a protein called pyrin, which is an important active component of the inflammasome. The relationships between the mutation sites of *MEFV* and IgAV phenotypes are still inconclusive (148–150). For example, *MEFV* E148Q polymorphism (G >C) increases the risk of IgAV and is associated with joint involvement (151). Other non-HLA signals are mainly genes implicated in vascular inflammatory pathways. Polymorphism in enzyme encoding genes such as *C1GALT1* may affect the synthesis of Gd-IgA1 and interfere with the IgA1 glycosylation process (152, 153). Moreover, polymorphism in genes encoding cytokines such as IL-1 and IL-8, chemokines, TLRs, vascular endothelial growth factor, renin–angiotensin system, and nitric oxide synthase is also associated with the risk of IgAV (139, 154–159).

### 3.7 Epigenetics

Epigenetic regulation, including DNA methylation, histone modifications, and non-coding RNAs, has emerged as one of the key mechanisms regulating gene expression and cell development or differentiation, and also plays a role in immune diseases (160, 161). The “histone code” is a set of post-translational modifications, including acetylation, methylation, and phosphorylation, that can confer precise functional properties to specific gene segments (162). Cross-phenotype analysis of immunochip data identifies KDM4C, the gene encoding histone demethylase, as a common risk locus for multiple vasculitis in children (163). Luo et al. observed aberrant histone modifications in peripheral blood mononuclear cells (PBMCs) from patients with IgAV, and the pronounced increase of H3 acetylation and H3K4 methylation both reached the genome-wide level, higher in patients with nephritis than in non-nephritis patients (164). Due to the IL-4 promoter and enhancer regions of CD4^+^ T cells enriched in H3 acetylation and H3K4me3, the Th1/Th2 balance is further disrupted in IgAV patients, leading to a Th2 bias and promoting the development of disease. Advances in the field of epigenetic regulation have deepened the molecular mechanisms underlying IgAV pathogenesis and may prove novel insights for the treatment of IgAV.

### 3.8 Biomarkers

The prognosis of IgAV largely depends on the incidence of serious gastrointestinal, pulmonary, and neurological complications in the immediate period and the severity of renal involvement. The current studies on biomarkers also mainly target these two aspects. Renal biopsy is the gold standard for assessing renal injury in IgAVN, but it is invasive and not easily monitored dynamically. Searching for non-invasive biomarkers with high sensitivity and specificity is of clinical importance for early identification, timely intervention, dynamic monitoring, and evaluation of disease efficacy and prognosis.

The main candidate biomarkers for severe gastrointestinal involvement of IgAV include plasma coagulation factor XIII activity and neutrophil/lymphocyte ratio (NLR) (165–167). Both are easy to obtain clinically, and active measures can be taken for biomarker-positive patients. Factor XIII catalyzes the cross-linking of fibrin and plays an important role in clot formation and wound healing. IgAV causes a decrease in factor XIII activity during its acute phase, presumably due to degradation by proteolytic enzymes released during inflammation, in proportion to inflammatory damage (168, 169). Also, these biomarkers provide new therapeutic targets in the treatment of IgAV. Case reports indicate that coagulation factor XIII infusion can improve gastrointestinal symptoms (170, 171).

Nephritis is a major long-term complication of IgAV, where a prospective study in Finland showed that age older than 8 years and recurrence are risk factors for the development of nephritis (172). In general, the prognosis in children is better than in adults (173). With the advanced technology of biological detection in recent years, serum and urine biomarkers of kidney injury in IgAVN have constantly been discovered. Urine markers can be divided into two main categories: preclinical and clinical. Williams et al. stated that the most dominant preclinical urine biomarkers of IgAVN are kidney injury molecule-1 (KIM-1), MCP-1, β-N-acetylglucosaminidase (NAG) and urinary angiotensinogen (UAGT) (174). The first three also correlate with disease severity. KIM-1 and NAG are usually upregulated after renal tubular damage and thought to be associated with tubulointerstitial inflammation. The urinary concentrations decrease when the disease is in remission, showing a relationship with disease activity (175, 176). Furthermore, the accepted clinical urine biomarkers are urine protein concentrations, including 24-hour urinary protein, the urinary protein to urinary creatinine ratio, and others. A prospective cohort study of adults identified other urine markers for predicting IgAVN, including IgA, IgG, IgM, netutrophil gelatinase-associated lipocalin (NGAL), IL-1β, IL-6, IL-8, IL-10, IgA-IgG, and IgA-sCD89 complexes, and it is worth pointing out that urinary IgA levels at the onset of illness may predict poor renal outcome, which can possibly serve as a routine marker for IgAVN (51). In addition to the above urine biomarkers, serum Gd-IgA1 and specific antibody levels, apolipoprotein M, matrix metalloproteinase-9 (MMP-9), red blood cell distribution width, pentraxin 3, alpha-smooth muscle actin, and c-Met have also been reported to be associated with the risk of IgAV nephritis (37, 177, 178). However, the predictive values for circulating IgA level are divergent across studies. The findings of Hočevar et al. showed that patients with elevated serum IgA seem to have a higher risk of renal involvement, while a study on biomarkers of childhood IgAV nephritis showed no significant differences in the serum IgA level comparing cases without nephritis (176, 179). On the other hand, Tan et al. found that elevated serum bilirubin levels might be related to favorable renal outcomes (180).

What calls for special attention is that although biomarkers can serve as warnings, there is no effective means to identify which patients are likely to suffer irreversible kidney damage at disease onset. Kidney biopsy is vital to assess disease activity and prognosis and determine therapy selection. It is recommended that all patients be monitored for at least 6 to 12 months, and monitoring can be prolonged in patients at high risk of nephritis (4, 181). For those with continuously aggravated symptoms or no-remission after treatments, especially patients with impaired eGFR, severe or persistent proteinuria (UP : UC ratio >250 mg/mmol for 4 weeks; UP : UC ratio >100 mg/mmol for 3 months; UP : UC ratio >50 mg/mmol for 6 months), timely renal biopsy and active intervention should be performed (4). It is important for clinicians to integrate the clinical features with different histological findings as well as biomarkers of IgAV patients and observe patients at regular intervals in order to better decide the time for initiating glucocorticoids or other immunosuppressive therapies and improve the prognosis.

## 4 Conclusion

IgAV is a multifactorial vascular disease, and the epidemiological features highlight the influences of genetic and environmental factors. The pathogenesis of IgAVN is similar to the “four-hit” hypothesis of IgAN, in which the abnormal immune response of the body is activated by precipitating events such as infection, leading to overproduction of galactose-deficient IgA1 and the formation of an immune complex containing Gd-IgA1, which is then deposited in the kidney and promotes the development of IgAVN. Given these shared mechanistic foundations, experimental targeted treatments for IgAN are likely to be applied to IgAV. Another mainstream pathogenesis hypothesis is the “new multi-hit” model, in which the binding of IgA to FcαRI induces the migration and activation of neutrophils and causes systemic vascular inflammation, which better elucidates the extrarenal involvement mechanism of IgAV. In-depth exploration of IgAV pathogenesis is of great significance for disease prevention, prognosis improvement, and precise treatment at the molecular level. Nevertheless, the pathogenic mechanisms of IgAV are far from being completely understood, and the relationship between IgAV, IgAVN, and IgAN has not been fully clarified. Constant original research and more rigorous evidence are needed to reveal the essential and core pathogenesis of IgAV and IgAVN.

## Author contributions

LX drafted the manuscript and designed the figure. YL and XW revised the manuscript. All authors listed have made a substantial, direct, and intellectual contribution to the work and approved it for publication.

## Funding

This work was supported by grant from the National Key R&D Project (grant no. 2021YFC2702004).

## Conflict of interest

The authors declare that the research was conducted in the absence of any commercial or financial relationships that could be construed as a potential conflict of interest.

## Publisher’s note

All claims expressed in this article are solely those of the authors and do not necessarily represent those of their affiliated organizations, or those of the publisher, the editors and the reviewers. Any product that may be evaluated in this article, or claim that may be made by its manufacturer, is not guaranteed or endorsed by the publisher.
